# Artificial Neural Networks in Mammography Interpretation and Diagnostic Decision Making

**DOI:** 10.1155/2013/832509

**Published:** 2013-05-26

**Authors:** Turgay Ayer, Qiushi Chen, Elizabeth S. Burnside

**Affiliations:** ^1^H. Milton Stewart School of Industrial and Systems Engineering, Georgia Institute of Technology, 765 Ferst Dr., Atlanta, GA 30332, USA; ^2^Department of Radiology, University of Wisconsin Medical School, E3/311, 600 Highland Avenue, Madison, WI 53792-3252, USA

## Abstract

Screening mammography is the most effective means for early detection of breast cancer. Although general rules for discriminating malignant and benign lesions exist, radiologists are unable to perfectly detect and classify all lesions as malignant and benign, for many reasons which include, but are not limited to, overlap of features that distinguish malignancy, difficulty in estimating disease risk, and variability in recommended management. When predictive variables are numerous and interact, ad hoc decision making strategies based on experience and memory may lead to systematic errors and variability in practice. The integration of computer models to help radiologists increase the accuracy of mammography examinations in diagnostic decision making has gained increasing attention in the last two decades. In this study, we provide an overview of one of the most commonly used models, artificial neural networks (ANNs), in mammography interpretation and diagnostic decision making and discuss important features in mammography interpretation. We conclude by discussing several common limitations of existing research on ANN-based detection and diagnostic models and provide possible future research directions.

## 1. Introduction

Breast cancer is the most common nonskin cancer and the second leading cause of cancer deaths among American women [1]. About one in eight American women are projected to develop breast cancer in their lives [2]. The American Cancer Society (ACS) estimates that 288,130 women were diagnosed with breast cancer and 39,520 died from this disease in 2011 [3].

Unfortunately, there is no foolproof method to prevent breast cancer. However, when detected early, the disease is often effectively curable. For example, 5-year survival rate increases from 27% to 98% when breast cancer is detected in an early stage [3]. That is why there is an intense interest in screening modalities for early detection.

Mammography, a low-dose X-ray procedure for visualizing the internal structure of the breast, is the most effective means to date for early detection of breast cancer [4]. Mammograms can detect masses, tiny deposits of calcium referred to as microcalcifications, and other subtle changes that may indicate cancer. Early diagnosis through screening mammography is the most effective means of decreasing the death rate from breast cancer. Randomized trials have shown that the use of screening mammography in the general population reduces breast cancer mortality by at least 24 percent [5]. It is estimated that more than 20 million mammograms are performed in the US annually and approximately 70% of women over age 40 have had a mammogram in the last two years [6, 7].

All mammograms are overseen and interpreted by radiologists. Subspecialty radiologists who are experts in the field often have fellowship training in mammography and read these studies exclusively. Community radiologists, who read the majority of mammograms in the context of a diverse general practice, on the other hand, have lower rates of cancer detection and higher rates of biopsy [8]. It is reported that in the US, only about 20% of women who have biopsies turn out to have cancer [9]. While only about 3.5% of abnormal screening mammograms interpreted by community radiologists reveal cancer, subspecialty radiologists have a significantly higher positive predictive value (PPV) [8]. Community radiologists also have a lower sensitivity resulting in missed breast cancers. While community radiologists detect about 3.0 breast cancers per 1,000 screening mammograms, subspecialty radiologists detect significantly more: about 5.3 cancers per 1,000 mammograms [10]. Furthermore, the US as a whole appears to have different decision thresholds than other countries. Smith-Bindman et al. [11] report that although cancer detection rates are identical in the US and in the UK, radiologists in the US declared many more mammogram results uncertain or suspicious compared with their British counterparts. As a result, American women with and without cancer underwent at least double the number of followup tests, like biopsies. 

The American College of Radiology (ACR) has been working on addressing these issues by attempting to standardize mammography reporting, reduce confusion in breast imaging interpretations, and facilitate outcome monitoring. For example, the ACR has developed a lexicon, the breast imaging reporting and data system (BI-RADS), which standardizes mammogram feature distinctions and the terminology used to describe them [12]. The BI-RADS lexicon, which includes the descriptors that can predict benign or malignant disease, is intended to guide radiologists and physicians in the breast cancer decision making process to facilitate patient management. Furthermore, the results can be compiled in a standard format that permits the collection, maintenance, and analysis of demographic, mammographic, and outcomes data.

Although general rules for discriminating malignant and benign lesions exist, radiologists are unable to classify all lesions as malignant and benign, as the successful diagnosis requires systematic search patterns using numerous factors in the presence of noise in images [13]. When predictive variables are numerous and interact, ad hoc decision making strategies based on experience and memory, the only viable method for radiologists, may lead to errors [14] and variability in practice [11, 15]. That is why there is intense interest in developing tools that can calculate an accurate probability of breast cancer to aid in decision making [16–18].

To improve the accuracy of mammography interpretation and aid in detection and diagnosis of abnormalities, several computer-aided detection (CAD) and computer-aided diagnostic (CADx) tools have been developed. The integration of computer models to help radiologists increase the accuracy of mammography examinations in diagnosis [19–23] has gained increasing attention since the last two decades. CAD and CADx models may help radiologists in the detection and discrimination of lesions as benign and malignant by providing objective information, such as the risk of breast cancer [24]. In this paper, we provide an overview of one of the most commonly used models, artificial neural networks (ANNs), in CAD and CADx for mammography interpretation and biopsy decision making and discuss important features in breast cancer diagnosis. We present a list of the articles described in this study in Table 1.

## 2. ANN Models in Breast Cancer Detection and Diagnosis

**Figure 1:**
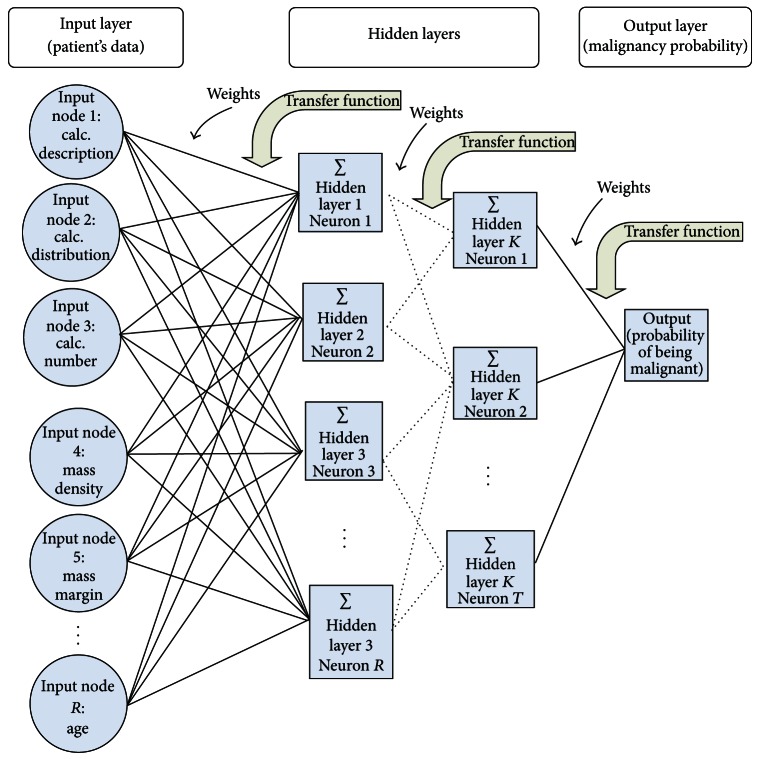
Inputs to the network are lesion descriptors and family history of the patient. Nodes at each layer are connected to the nodes at the succeeding layer by weighted arcs. Each hidden node in the first hidden layer performs a nonlinear weighted sum of all input values. The outputs of the last hidden layer are then similarly combined to the output layer. The single output value shows the probability of the lesion being malignant.

ANNs are computer models that have the ability to duplicate aspects of human intelligence while incorporating the processing power of computers and are thus capable of processing a large amount of information simultaneously by learning from previous cases [25]. ANNs have many desirable properties that make them well suited for medical decision making. ANNs are capable of “learning” complicated patterns from data that are difficult for humans to identify [26]. They can also often overcome ambiguous and missing data [27] and provide accurate predications [28, 29]. The structure of a generic ANN model built for aiding in mammography interpretation is presented in Figure 1. The ANN models built for aiding in mammography interpretation typically take patients demographic risk factors (such as age and a family history) and mammographic findings (such as mass or calcification variables) as inputs and estimate the corresponding breast cancer risk to aid in biopsy decision.

Microcalcifications are one of the major indicators of breast cancer. A large proportion, 30%–50%, of breast cancers demonstrate microcalcifications on mammography, and 60%–80% of cancers exhibit microcalcifications on histologic examination [30, 31]. Identifying microcalcifications, which range in size between 0.1 and 1 mm, is a difficult detection task for radiologists [31, 32]. Furthermore, distinguishing between malignant and commonly occurring benign microcalcifications is challenging.

 There are two different ways of using ANNs to aid in mammography interpretation. The first approach is to apply the classifier directly to the region of interest (ROI) image data. As a second approach, ANNs can also learn from the features extracted from the preprocessed image signals. Below, we summarize some of the noteworthy studies that took the first approach.

Stafford et al. [33] developed a committee of three-layer ANNs to examine digital mammograms after image preprocessing. These ANNs were trained and tested on 256 mammograms and transformed the original ROI images into output images such that each pixel was assigned a value between 0 and 1. The committee consisted of four ANNs, each with expertise on identifying microcalcifications within a certain size range. In particular, the four ANNs were built by using the microcalcification samples with size ranges of 50–250 *μ*m, 100–500 *μ*m, 200–1,000 *μ*m, and 400–2,000 *μ*m, respectively. The committee took the highest output among these four ANNs (the winner-take-all rule) as the output for each pixel. The full system was tested on microcalcifications of size ranging from 50–2,000 *μ*m. The committee reached 84% sensitivity at 75% specificity.

Zhang et al. [30] developed a novel neural network to identify whether an ROI included more than a pre-underspecified number of microcalcifications. In this proposed neural network model, a subsequent layer did not depend on the location patterns in the preceding layer, a special structure called the shift-invariant property. Therefore, the result of the shift-invariant ANN (SI-ANN) did not depend on the location information in the input ROI images. If a classical back-propagation ANN (BP-ANN) was used instead, then the locations of the microcalcification clusters implicitly had to be encoded as the inputs of this neural network. The performance of the SI-ANN was evaluated using a database of 168 ROIs of 55 × 55 pixels and various network configurations. Using this method, the highest area under the ROC curve (*A*_*Z*_) of 0.91 ± 0.02 was achieved. The neural network was able to eliminate approximately 55% of false positive ROIs without missing any of true-positive ROIs. Furthermore, the SI-ANN showed a superior performance over the classic BP-ANN [34].

Chan et al. [35] investigated the effectiveness of a convolutional neural network (CNN) in detecting microcalcifications on mammograms. The CNN was different from the classic ANN in its structure where nodes in hidden layers were organized in groups. In the CNN, the same values were enforced for the weights connecting the nodes in groups of subsequent layers, which enabled the neural network to incorporate the neighborhood information around each pixel on mammograms during the training process. The output of the CNN was a decision score. The performance of the CNN was evaluated on a data set of 52 mammograms. The average *A*_*Z*_ was 0.9, which was substantially robust to different network configurations. The CNN further reduced the number of false-positive clusters per image by more than 70% at all true-positive rates.

As a second approach, instead of learning directly from images, ANNs can also learn from the features extracted from preprocessed image signals. Several ANN applications for reducing false-positive (FP) cases in microcalcification detection followed this approach [36–39]. Among these studies, Nagel et al. [36], for example, built an ANN for identifying microcalcifications based on five extracted features: area, contrast, first moment of the power spectrum, mean pixel value, and edge gradient. This ANN was trained on 39 mammograms, and its output represented the likelihood of being a microcalcification. A feature-wise threshold was computed based on the training data to minimize the number of false positives while maintaining a high enough true-positive (TP) rate. For comparison purposes, a rule-based method of FP reduction was also built. The average number of FPs per image were 1.9 for the rule-based method, 1.6 for the ANN, and 0.8 for the combined method at a sensitivity of 83%, when they were evaluated on an independent test set of 50 mammograms. 

Following the detection of microcalcifications, radiologists should decide whether to biopsy or not. This decision relies on the ability of the radiologist to accurately differentiate benign and malignant features. To aid in biopsy decision making, several ANN-based CADx models based on radiologists' observations have been developed since 1990s [40–43].

As an alternative to feature extraction based on radiologists' observations, algorithms were developed to automatically extract features from digital mammography images. These automatically extracted features can be used as input to feed the CADx models. Chan et al. [44] provided a comprehensive summary of such methods. Jiang et al. [45] first integrated the computerized feature analysis and discrimination. Only the initial identification of microcalcification clusters was performed by radiologists. Based on eight morphological features extracted from the image, the ANN identified 100% of the malignant and 82% of the benign cases. The accuracy was significantly higher than that of five radiologists without computer aid (*P* = 0.03). In a follow-up study, Jiang et al. [46] compared the automated discrimination methods and routine clinical performance by ten radiologists using an ROC analysis. The ROC index *A*_*Z*_ increased from 0.61 without aid to 0.75 with computer aid (*P* < 0.0001). This improvement was also reflected in sensitivity (73.5% to 87.4%) and specificity (31.6% to 41.9%). In the method proposed by Huo et al. [47], mass regions were identified automatically and then features related to the margin and density of each mass were extracted. The results showed that the discrimination performance of the ANN (*A*_*Z*_ = 0.94) was slightly better than that of an experienced mammographer (*A*_*Z*_ = 0.91) and significantly better than the average radiologists (*A*_*Z*_ = 0.81, *P* = 0.13). Similarly, in Kallergi [22], features were automatically extracted from digital images by detection/segmentation methods. The ANN based on fourteen morphological (for individual calcifications) and distributional (for the clusters) descriptors was shown to achieve high sensitivity and specificity (100% and 85%), and be robust against false positive signals.

In addition to morphological features extracted from mammography images, texture features were also used to feed ANNs in classifying malignant and benign microcalcifications, such as in the study by Chan et al. [48]. In this study, thirteen texture features were derived from spatial grey level dependence (SGLD) matrices, which were constructed from the background-corrected ROIs. Several representative subsets of features were evaluated by a stepwise procedure. The feature set consisting of six features achieved the highest accuracy (*A*_*Z*_ = 0.88). The sensitivity was 100% at a specificity of 24% when decision threshold was set to 0.85. The results of this study showed that computerized methods were able to capture the changes of texture features in malignant, which were not visually apparent on mammograms. 

Obviously, mammographic features are not the only considerations for physicians in breast cancer diagnosis. Other relevant findings, such as a patient's medical history and clinical factors, can also be informative for a successful diagnosis. Baker et al. [41] built an ANN model based on ten descriptors from breast imaging reporting and data system (BI-RADS) and eight features of patients' medical history such as age, personal and family history of breast cancer, and menopausal status. In this study, the specificity of the ANN was significantly greater than that of radiologists (62% and 30% at 95% sensitivity, *P* < 0.01). Later, Lo et al. [42] observed similar findings and showed that age was a strong diagnostic predictor in the retrospective evaluation of the follow-up study. Considering age together with seven BI-RADS findings in the ANN significantly enhanced the discrimination performance measured in *A*_*Z*_ (*P* = 0.028). 

In addition to mammographic features, some studies built ANN models that also considered sonographic features. Among them, the first one, Jesneck et al. [49] examined 803 breast mass lesions (296 malignant and 507 benign) from 737 patients. To assess the discrimination performance, ROC analysis was used in a training, validation, and retest scheme. Results showed that the ANN model achieved a high performance (*A*_*Z*_ = 0.92 ± 0.01), and consideration of sonography variables improved the performance.

**Figure 2:**
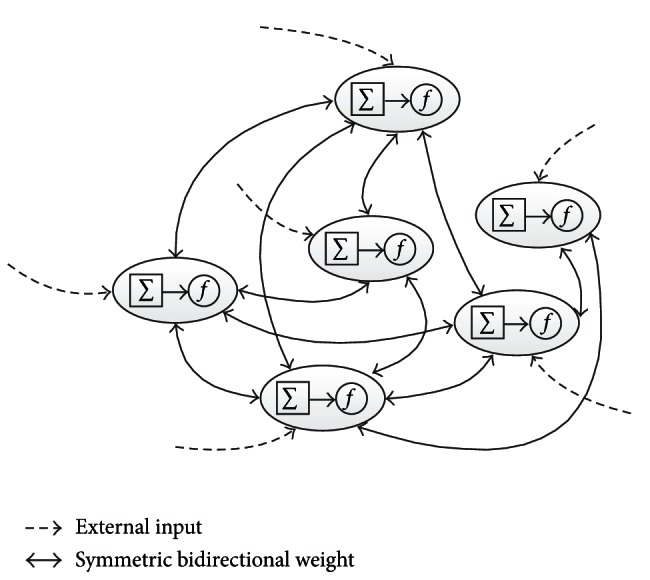
Neurons are organized in a non-hierarchical structure in constraint satisfaction neural network (CSNN). Each neuron is assigned a value (activation level). These values represent the network state. Inputs to each neuron include both the external input and the activation levels of other neurons connected by the bidirectional symmetric weights. The activation levels are updated by passing the weighted sum of input values through a transfer function. The training is terminated when the network achieves a globally stable state with all constraints satisfied.

Although ANNs have been successful in mammographic diagnosis, they have often been regarded as black box since they do not provide much clinical intuition. To overcome this limitations, Tourassi et al. [50] proposed an innovative ANN, the constraint satisfaction neural network (CSNN), as illustrated in Figure 2. An appealing property of this nonhierarchical and flexible CSNN model was the capability to discover trends and hidden associations (e.g., to identify the risk factors) and extract decision rules. As inputs, 10 mammographic and six patient clinical features of 500 breast lesions (174 malignant and 326 benign) from BI-RADS database were used. Based on a 50%-50% cross-validation scheme, the ROC index *A*_*Z*_ was shown to be 0.84 ± 0.02, which was comparable with the performance of a classic ANN [42]. Later, Tourassi et al. [51] validated this approach using a larger testing data set of additional 1,030 cases. 

In addition to improving diagnostic accuracy, ANNs have also been useful in reducing variability in radiologists' interpretations. In the literature, significant variability in radiologists' interpretations has been reported. For example, a recent study by Beam et al. [52] showed that the sensitivity of mammography ranged from 59% to 100% and specificity ranged from 35% to 98%, depending on the reading radiologist. To reduce this interobserver variability, Jiang et al. [46], for the first time, presented evidence for the ability of an ANN model to reduce variability of mammography interpretation among radiologists. In another study, Jiang et al. [53] assessed the variability in interpretation among radiologists with and without an ANN model. The ANN estimated the likelihood of malignancy, and ten radiologists were instructed in how to utilize the output of the ANN. The findings of this study verified that ANNs were not only useful for improving diagnostic accuracy but also for decreasing variability in mammography interpretation. In particular, they showed that (1) the range in sensitivity was reduced from 35% to 26% and the standard deviation (SD) of *A*_*Z*_ reduced by 46% (from 0.056 to 0.030); (2) on average, complete agreements were achieved in 33 (32%) cases with computer aid compared with 13 (13%) cases without the aid (*P* < 0.001), and the occurrence of conflicting was reduced from 43 (41%) cases to 28 (27%) cases (*P* = 0.02); (3) substantial disagreements in recommendation (biopsy versus routine follow-up, measured by pairwise frequency and per-patient frequency (see [15]), were reduced significantly with computer aid for all cases and for cancer cases only (*P* < 0.04). 

The results of mammography are often conveyed as positive or negative. In reality, however, the result of any test that is imperfect would ideally be expressed in terms of a post-test probability of disease which would help an individual better understand his or her personal risk given the sensitivity and specificity of the test. Recall that the output of an ANN is often a probability indicating the similarity of the test case to the malignant or benign findings. Then, a preset threshold value is used to determine whether the test case is malignant or benign. In this regard, ANNs can also be viewed as risk assessment models. However, most ANN studies in the literature have only focused on discrimination but did not consider calibration. Orr [54] explored the value of quantifying the risk of malignancy using an ANN. A standard back-propagation network with a single hidden layer was trained and tested on a dataset of size 1,288 (75% for training and 25% for testing). The ROC index *A*_*Z*_ of the ANN in the test set was 0.89, which was significantly better than that of the physicians alone (*A*_*Z*_ = 0.86, *P* < 0.01). In a retrospective examination of the training data, the author observed that among the patients with an ANN output of 0, none had cancer, and for those with an output greater than 0.75, 71% of them had cancer. To assess the risk stratification capability of ANN (i.e., calibration), patient data were divided into four quartiles, four subgroups of almost equal size based on the magnitude of the ANN output, where those in the lowest quartile had minimal risk of malignancy. Results showed that the risks of cancer were well separated among the four subgroups (2/391 = 0.5%, 7/272 = 2.6%, 37/341 = 10.9%, and 139/295 = 47.1%, resp.). 

Risk estimations provided by ANNs could provide useful information for physicians for a successful diagnosis, risk stratification, and risk communication. As noted by Cook [55], a comprehensive evaluation of such models should include both discrimination and calibration. The discrimination ability represents the capability to separate the malignant findings from the benign ones, as measured by ROC index *A*_*Z*_, sensitivity and specificity. Discrimination assessment is commonly used as we see in studies reviewed above. However, discrimination measures cannot assess how well the predictions agree with the actual observations, which needs to be evaluated via the model calibration. The purpose of calibration is to improve the accuracy of risk prediction by estimating the absolute risk of cancer. A well-calibrated model means that the predicted risks match the observed risks within each subgroup [56]. However, unlike discrimination, calibration did not receive much attention in performance assessment of the existing ANN models. 

There is a tradeoff between discrimination and calibration, and perfect calibration and discrimination cannot be achieved simultaneously in clinical practice [57–59]. Several studies have shown that given a perfectly calibrated risk estimation model, the ROC index *A*_*Z*_ varied with the distribution of the observed risk in the population. 

Ayer et al. [43] revisited the use of ANN models in breast cancer risk estimation and assessed both discrimination and calibration. On a large data set consisting of 62,219 consecutive mammography findings, the risk prediction was obtained using 10-fold cross-validation. The ANN model achieved an *A*_*Z*_ of 0.965, which was significantly higher than that of the radiologists, 0.939 (*P* < 0.001). The calibration of the ANN was assessed by the Hosmer-Lemeshow (H-L) goodness-of-fit statistic test. The H-L statistic was 12.46 (*P* > 0.1, df = 8), which indicated a good match between the risk estimates and the actual malignancy prevalence.

In clinical practice, missing data is a common problem [51]. Obviously, incomplete inputs may have an impact on the prediction accuracy of a trained ANN. Markey et al. [27] investigated the impact of missing data in classifying testing data on ANNs. The ANNs were trained with complete data and tested on a dataset with missing components. Four levels of missing data (10%, 20%, 30%, and 40%) were tested in a back-propagation ANN (BP-ANN) and a CSNN model. For the BP-ANN, missing values were (1) replaced with zeros, (2) replaced with mean value from the training set, and (3) imputed by using a multiple imputation procedure. The results showed that the replacing of the missing values with zeros was not very efficient and could lead to misleading results. The decrease of *A*_*Z*_ was significant (*P* < 0.01) even with only 10% missing data (0.84 ± 0.03) compared with the complete data (0.94 ± 0.01). The other two methods were shown to be more accurate and efficient. Their findings showed that with data imputation, the models achieved reasonable performance for up to about 30% missing data. 

Imbalanced data presents another challenge to ANN development, testing, and performance. A data set is considered imbalanced if the number of instances of one class is significantly smaller than that of the other class. In the context of breast cancer, the proportion of patients with breast cancer is significantly lower due to the actual prevalence of the disease. Mazurowski et al. [60] showed that this influence could significantly reduce the performance of an ANN. In general, two methods, undersampling and oversampling, are commonly used to compromise data imbalance. Undersampling randomly chooses samples from the majority class so that the size of the majority class is similar to that of the minority class. Oversampling, on the other hand, will randomly duplicate or interpolate the samples from the minority class to mitigate this imbalance. Mazurowski et al. [61] investigated the effects of imbalanced data on the discrimination performance for a classic ANN. A database consisting 1,005 biopsy-proven masses (370 malignant and 645 benign) collected at the Duke University Medical Center was used to compare the effects of oversampling and undersampling. This study verified the detrimental effects of the class imbalance in training dataset and showed that oversampling in general achieved a higher ROC performance compared with undersampling. 

## 3. Discussion and Conclusions

Several studies have verified that ANNs have the potential to successfully aid in mammography interpretation and breast cancer diagnosis. However, for successful applications of ANNs, both advantages and disadvantages of these models should be well understood and be carefully considered by researchers and the end users. Advantages and disadvantages of ANNs have been previously discussed in several studies in the literature (see, e.g., [62, 63]). To summarize, the advantages of ANNs include the ease of model building, the capability in capturing the interactions between predictors, and ability to consider complicated nonlinearities between predictors and outcomes (Table 2). 

Besides the advantages, ANNs have several disadvantages as well. In medical practice, the clinical insights obtained from the prediction models obviously play an important role. As Tu [63] noted, ANNs however suffer from the limited capability to explicitly explain the causal relationships between predictive factors and outcomes, which is probably the most major drawback. Another drawback is that a well-trained model would be difficult to share with other researchers. This is because the knowledge discovered from the data is all encoded into a huge weight matrix, which is difficult to interpret and share. Furthermore, the complexity of the model structure in ANNs makes it more prone to overfitting, the case where the network overlearns and mimics the training dataset but performs poorly when presented to an external dataset. Ayer et al. [25] also noted the need for confidence intervals, which are, unlike statistical methods, not straightforward to obtain from ANN models. 

## 4. Future Research in ANNs for Breast Cancer Detection and Diagnosis

There is a growing interest in developing successful ANN models for breast cancer detection and diagnosis, due to high computational power and practical use of ANNs. However, many studies in the literature share some common limitations, which make their applications limited. As noted by Schwarzer et al. [64], the most common major limitations include (1) lacking a comprehensive assessment of the discrimination accuracy, (2) overfitting, and (3) the complexity issues. First, most studies in the literature do not evaluate the performance of the trained ANNs using an independent test set. If testing the model on an independent dataset is not feasible due to data limitation or other concerns, at least cross-validation should be done to minimize the potential bias. However, many studies lacked such evaluations and as a result, in most cases, the error rates were dramatically underestimated. Second, most studies did not pay close attention to overfitting. The generalizability of the neural networks substantially depends on the number of hidden nodes in the hidden unit. When they are too few, the network is limited in its capability of representing the causal relationships. On the other hand, when they are excessive, the network is prone to overfitting. Many studies in the literature reported the use of very large the number of hidden nodes as compared with the size of the training data but did not assess whether overfitting occurred. Lastly, in many studies, the computational complexity of the ANN was not properly reported. Some measured the complexity only using the number of input units which would underestimate the computational complexity. Properly reporting the complexity of an ANN model is important because the computational power as well as many potential problems such as overfitting are closely related to the complexity of the model. The future studies in this domain should carefully consider and overcome these limitations for successful applications of ANNs in mammography interpretation.

## Figures and Tables

**Table 1:** Summary of ANN studies in mammography interpretation and diagnostic decision making.

Study	Type	ANN structure	Input	Dataset and training/testing strategy	Results and findings
Stafford et al. (1993) [33]	CADe	A committee of four three-layer BP-ANNs	Pixel information	167 mammograms with pathologies and 89 without pathologies. 50% for training and 50% for testing.	Test on 20 out of 128 mammograms covering microcalcification size-range of *50*–*250 *μ*m*: 0.9% FP at 85% TP, 2.4% FP at 100% TP. *50–2,000 *μ*m*: 25% FP at 84% TP, 40% FP at 100% TP.

Zhang et al. (1994) [30]	CADe	The Shift-Invariant ANN (SI-ANN)	Pixel information	168 ROIs from 34 digitized mammograms. 50%-50% cross-validation.	ROC index: A_Z_ = 0.91 ± 0.02, 45% FP at 100% TP.

Chan et al. (1995) [35]	CADe	The Convolution Neural Network (CNN)	Pixel information	52 mammograms *Group 1*: 110 TP and 116 FP. *Group 2*: 108 TP and 116 FP. Two-fold cross-validation.	*ROC index*: A_Z_ = 0.9. *FP rate*: 0.1 cluster per image at 100% TP (for obvious cases), 1.5 cluster per image at 90% TP (for average and subtle cases).

Nagel et al. (1998) [36]	CADe	SI-ANN	Features extracted from image	196 TPs and 1,252 FPs. Leave-one-out cross-validation.	*The number of FPs per image at 83% TP*: 1.6 for ANN, 0.8 for ANN and rule-based method. *Average ROC index*: A_Z_ = 0.85 (stdev = 0.04) for ANN, A_Z_ = 0.64 (stdev = 0.07) for ANN + rule-based method (*P* = 0.014).

Wu et al. (1992) [34]	CADe	BP-ANN	Pixel information	56 positive, 56 negative, and 56 FP ROIs, respectively. 50%-50% cross-validation.	*For individual microcalcifications*: A_Z_ = 0.71. *For clustered microcalcifications*: A_Z_ = 0.85; 50% FP at 95% TP.

Jiang et al. (1996) [45]	CADx	BP-ANN	Computer-extracted morphological features	40 malignant and 67 benign cases from 100 images. Leave-one-out cross-validation.	Identified 100% malignant and 82% of the benign cases. Significantly better than radiologists without computer aid (*P* = 0.03).

Jiang et al. (1999) [46]	CADx	BP-ANN	Computer-extracted morphological features	46 malignant and 58 benign cases. Leave-one-out cross-validation.	*By 10 radiologists*: A_Z_ = 0.61, sensitivity = 73.5%, specificity = 31.6%. *With aid of ANN*: A_Z_ = 0.75 (*P* < 0.0001), sensitivity = 87.4%, specificity = 41.9%.

Huo et al. (1998) [47]	CADx	BP-ANN	Morphological features characterizing margin and density	38 benign and 57 malignant cases from 65 patients. Leave-one-out cross-validation.	*ANN*: A_Z_ = 0.90, 19.2% specificity at 100% sensitivity. *Hybrid method (rule-based + ANN)*: A_Z_ = 0.94, 69.2% specificity at 100% sensitivity.

Kallergi (2004) [22]	CADx	BP-ANN	Morphological and distributional descriptors	50 benign and 50 malignant cases. Leave-one-out cross-validation.	A_Z_ = 0.98 ± 0.01, 85% specificity at 100% sensitivity.

Chan et al. (1997) [48]	CADx	BP-ANN	Texture features SGLD matrices	41 malignant and 45 benign cases from 54 patients. Leave-one-out cross-validation.	With best subset of features: *Mammogram-by-mammogram*: A_Z_ = 0.88, 24% specificity at 100% sensitivity. *Patient-by-patient*: 39% specificity at 100% sensitivity.

Baker et al. (1995) [41]	CADx	BP-ANN	BI-RADS lesion descriptors and medical history variables	133 benign and 73 malignant cases. Leave-one-out cross-validation.	*PPV*: 61% (ANN) versus 35% (radiologists). *ROC index*: A_Z_ = 0.89 ± 0.02 (ANN) versus 0.85 ± 0.03 (radiologists), *P* = 0.29. *Specificity*: 62% (ANN) versus 30% (radiologists) at 100% sensitivity (*P* < 0.01).

Lo et al. (1999) [42]	CADx	BP-ANN	BI-RADS lesion descriptors, age, and history variables	326 benign and 174 malignant cases. Leave-one-out cross-validation.	*Only BI-RADS features*: A_Z_ = 0.84 ± 0.02, 6% specificity at 100% sensitivity. *BI-RADS + age*: A_Z_ = 0.86 ± 0.02, 30% specificity at 100% sensitivity. *All features*: A_Z_ = 0.87 ± 0.02, 22% specificity at 100% sensitivity. Age variable significantly improves the A_Z_ (*P* = 0.028).

Ayer et al. (2010) [43]	CADx	BP-ANN	Demographic, mammographic features, and BI-RADS categories	510 malignant and 61,709 benign cases. 10-fold cross-validation.	A_Z_ = 0.965 (ANN) versus 0.939 (radiologists), *P* < 0.001. Specificity at 85% sensitivity: 94.5% (ANN) versus 88.2% (radiologists), *P* < 0.001.

Jesneck et al. (2007) [49]	CADx	BP-ANN	Mammographic features, sonographic features, and history features	296 malignant and 507 benign cases. 500 for training and validation, 303 for testing.	Training and validation set: A_Z_ = 0.92 ± 0.01, Testing set: A_Z_ = 0.91 ± 0.02.

Tourassi et al. (2003) [51]	CADx	CSNN	BI-RADS features, age and history	Training set: 174 malignant and 326 benign cases. Testing set: 358 malignant and 672 benign cases.	On training set: A_Z_ = 0.84 ± 0.02 On testing set: A_Z_ = 0.81 ± 0.02 CSNN is also capable to impute missing data.

Orr (2001) [54]	CADx and risk estimation	BP-ANN	Age and radiographic features	185 malignant and 1,103 benign cases. 490 for training and the rest for testing.	A_Z_ = 0.89 (surgeons) versus 0.86 (ANN), *P* = 0.004. ANN is possible for risk stratification.

CADe: computer-aided detection, CADx: computer-aided diagnosis, ANN: artificial neural network, BP-ANN: back-propagation artificial neural network, FP: false positive, TP: true positive, ROI: region of interest, SGLD: spatial grey level dependence, PPV: positive prediction value, BI-RADS: the breast imaging reporting and data system, CSNN: constraint satisfaction neural network, and SI-ANN: shift-invariant artificial neural network.

**Table 2:** Advantages and disadvantages of ANNs.

Advantage	Disadvantage
(i) Easy model building with less formal statistical knowledge required.	(i) Clinical interpretation of model parameters is difficult (black boxes).
(ii) Capable of capturing interactions between predictors.	(ii) Sharing an existing ANN model is difficult.
(iii) Capable of capturing nonlinearities between predictors and outcomes.	(iii) Prone to overfitting due to the complexity of model structure.
(iv) Users can apply multiple different training algorithms	(iv) Confidence intervals of the predicted risks are difficult to obtain.
	(v) The model development is empirical. Few guidelines exist to determine the best network structures and training algorithms.
